# Enhancement of Room Temperature Ethanol Sensing by Optimizing the Density of Vertically Aligned Carbon Nanofibers Decorated with Gold Nanoparticles

**DOI:** 10.3390/ma15041383

**Published:** 2022-02-13

**Authors:** Mostafa Shooshtari, Leandro Nicolas Sacco, Joost Van Ginkel, Sten Vollebregt, Alireza Salehi

**Affiliations:** 1 Department of Electrical Engineering, K.N. Toosi University of Technology, Tehran 1631714191, Iran; shooshtari@email.kntu.ac.ir (M.S.); salehi@kntu.ac.ir (A.S.); 2 Laboratory of Electronic Components, Department of Microelectronics, Technology and Materials (ECTM), Delft University of Technology, 2628 CD Delft, The Netherlands; l.n.sacco@tudelft.nl (L.N.S.); h.j.vanginkel@tudelft.nl (J.V.G.)

**Keywords:** carbon nanofiber (CNF), gas sensor, decorated CNF, fiber per unit area, Plasma-Enhanced Chemical Vapor Deposition (PECVD), aerosol printing

## Abstract

An ethanol gas sensor based on carbon nanofibers (CNFs) with various densities and nanoparticle functionalization was investigated. The CNFs were grown by means of a Plasma-Enhanced Chemical Vapor Deposition (PECVD), and the synthesis conditions were varied to obtain different number of fibers per unit area. The devices with a larger density of CNFs lead to higher responses, with a maximal responsivity of 10%. Furthermore, to simultaneously improve the sensitivity and selectivity, CNFs were decorated with gold nanoparticles by an impaction printing method. After metal decoration, the devices showed a response 300% higher than pristine devices toward 5 ppm of ethanol gas. The morphology and structure of the different samples deposited on a silicon substrate were characterized by TEM, EDX, SEM, and Raman spectroscopy, and the results confirmed the presence of CNF decorated with gold. The influence of operating temperature (OT) and humidity were studied on the sensing devices. In the case of decorated samples with a high density of nanofibers, a less-strong cross-sensitivity was observed toward a variation in humidity and temperature.

## 1. Introduction

Since the discovery of carbon nanofibers, this material has received a great deal of attention for the use in gas sensors. The primary reasons for this are their good electronic conductivity and high specific surface area [1]. Besides, the high adsorption ability of CNF towards various molecules makes it a good material for chemical gas sensors with high sensitivity and selectivity [2]. The use of vertically aligned CNF arrays with well-engineered properties and uniform diameter and length deposited by Chemical Vapor Deposition (CVD) was shown to provide a unique structure for gas sensing [3]. By adjusting the growth parameters in the CVD method, one can change the diameter, length, and the number of fibers per unit area [4,5,6,7,8]. Especially the latter is typically difficult to control in competing technologies like vertically aligned carbon nanotubes. To date, various synthesis parameters are proposed to grow CNFs, which may end up to material with different nanofiber densities [9,10]. While most attempts have been focused on synthesis techniques and potential applications of CNFs with certain densities, less attention has been paid to the effect of the number of fibers per unit area on the gas sensing mechanism of CNFs.

On the other hand, owing to their unique electrical and catalytic properties, metal nanoparticles (NPs) have emerged as a new class of compounds that are interesting in several fields including gas sensors. The behavior of the charge carriers in CNF has been the subject of the gas sensing mechanism [11]. Decorating CNF is known as an external controlling agent for the transportation of charge carriers [12,13]. It is expected that metal nanoparticles display a full range of reactivity with gas molecules and show promise for further functionalization for high analyte response by attaching specific receptors [14,15]. Additionally, the interaction between pristine CNT and volatile organic compound molecules is too weak; therefore, bare CNF has been demonstrated to be poor in responding to the presence of Volatile Organic Compounds (VOCs) [16]. In recent years, gas sensors constructed by noble metal NPs/CNFs nanohybrids have attracted interest to improve sensitivity [3]. Decoration of CNF with noble metal nanoparticles, which can be formed into crystalline nanoparticles and adsorbed onto fibers, has proven to be of great value for sensing ammonia, hydrogen sulfide, and carbon monoxide [3,13,17,18,19,20,21]. Here, the free electrons of the nanoparticles dope the CNFs. It has recently been demonstrated that a CNF and gold NPs composite can not only enhance and protect the intrinsic properties of the particles but also offer an effective approach to enhance the properties of gas sensors [20]. Such points, therefore, encourage us to experimentally investigate the sensitivity of CNF decorated with gold NPs, as an ethanol vapor gas sensor and investigate the effect of density variation on the decorated CNF ethanol vapor sensor.

In this work, we reported the synthesis of CNFs with different densities and decorated with Au NPs and their performance as a gas sensor. In recent works, the focus is mainly on the functionalization of CNF and the improvement of its sensitivity. There is a lack of research in the literatures in considering the effects of fiber numbers in area on gas sensing properties and, consequently, the effects of gold decorations on these kinds of sensors. In this research, we tried to investigate the effects of the density of nanofibers on the gas sensing mechanism and the effect of density on the factor of improvement of functionalized CNF. The results are reported in the form of SEM and TEM micrographs and gas response diagrams, which may be useful for researchers to determine the optimum CNF sensor configuration, particularly in the field of the gas sensor and materials.

## 2. Experimental

### 2.1. CNF Growth

The aligned CNF bundles were grown on 9 nm of a patterned nickel catalyst on a thermally oxidized silicon wafer by Plasma-Enhanced Chemical Vapor Deposition (PECVD). For this, an AIXTRON Blackmagic CVD reactor was used. The growth parameters were chosen according to [22]. In this method, at the pressure of approximately 9 mbar, 700 sccm of H_2_ is entered into the chamber, and the temperature is ramped up to 500 °C. Next, acetylene (C_2_H_2_) is added to the chamber. Various acetylene flows in the range of 5–25 sccm were used to achieve different densities of carbon fibers. After 20 min growth time with ignition of a 100 W pulsed-DC plasma, the reactor is cooled down in a N_2_ atmosphere.

### 2.2. Gold Nanoparticle Decoration

To decorate the CNT samples with gold nanoparticles we used a nanoparticle aerosol printing method. Aerosol routes are ideal for the production of high purity and controllable nanostructured materials [23]. In this study, we used aerosols generated by a spark discharge generator: the VSP G1, which is developed by VS Particle B.V. The device was equipped with Au electrodes with the purity of 99.999%; it also uses 1.5 l/min N_2_ as carrier gas. The generator settings were set at 1 kV and 8 mA. The generated aerosol was fed to a nanomaterial printer prototype developed by VS Particle B.V., which combines a programmable xyz-stage with an inertial impactor to direct-write nanoparticle deposits. The system was set to operate at <1 mbar pressure and room temperature. The resulting deposits form a nanoporous structure with a large surface area per unit mass, which makes it attractive to be used as sensors or catalysts. The deposit dimensions of a line or dot of nanoparticles vary with nozzle distance, writing speed, or deposition time. The nanoparticle size can be tuned by changing the generator settings. In this experimental study, gold nanostructures were printed at three different points on the samples at a distance of 2 mm from each other. The nozzle with a 0.3 mm diameter orifice was placed at a distance of 0.5 mm above the sample in the chamber with a pressure of 0.7 mbar. Finally, the printing time was set to about 5 s per dot.

### 2.3. Measurement Setup

Square samples with the dimension of 1 cm × 1 cm cut from a 4″ wafer were used. Then, gas sensing properties of the samples were investigated by applying 100 nm gold or platinum electrodes with an e-beam evaporator with shadow mask on the surface of samples and exposing them into the chamber with a predetermined analyte concentration. One mm wide electrodes were deposited in the form of two parallel strips at the end of the sample. As an adhesive layer between the silicon oxide and electrodes, titanium or tantalum, each with a thickness of 10 nm were used for gold and platinum, respectively. (The use of two contacts was to investigate the effect of electrodes on the gas sensing result. The final results of all samples are reported based on gold electrodes.) The humidity and temperature of the chamber were constantly monitored (ACS, discovery, E-series). Moreover, a conductive carbon paste was used for the electrical connections between the electrodes and wires. The sensor response is defined by:

(1) $$\mathrm{Sensor}\;\mathrm{response}=\frac{R_{g}-R_{a}}{R_{a}}\times 100\%,$$

**Figure 1:**
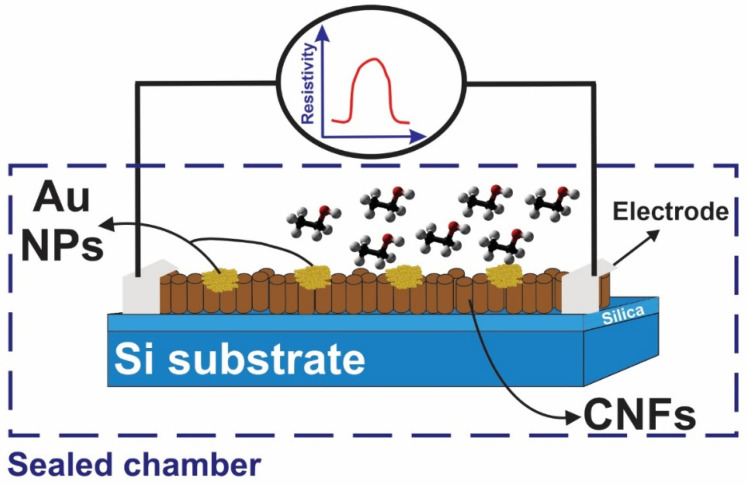
Schematic diagram of the gas testing equipment.

In which, *R_g_* and *R_a_* are sensor resistances at room condition (i.e., 25 °C temperature and 55% relative humidity) after exposure to the analyte and air, respectively. The measurement was performed by using the Keithley Source (2600B series) Measure Units by applying a 1 V DC voltage. The responses were obtained in the presence of ethanol vapors at different concentrations; this was performed by evaporating an exact amount of liquid ethanol, which was measured by a micro sampler. Ethanol concentrations were measured by dividing the mass of ethanol by the mass of air in the chamber. The method for the gas sensing tests were similar to previous research, and the reader is referred to refs. [24,25] for additional information. Figure 1 shows the schematic of a fabricated sensor placed inside the chamber and electronic measuring equipment connected to the sensor outside of the chamber.

## 3. Results and Discussion

### 3.1. Characterization

**Figure 2:**
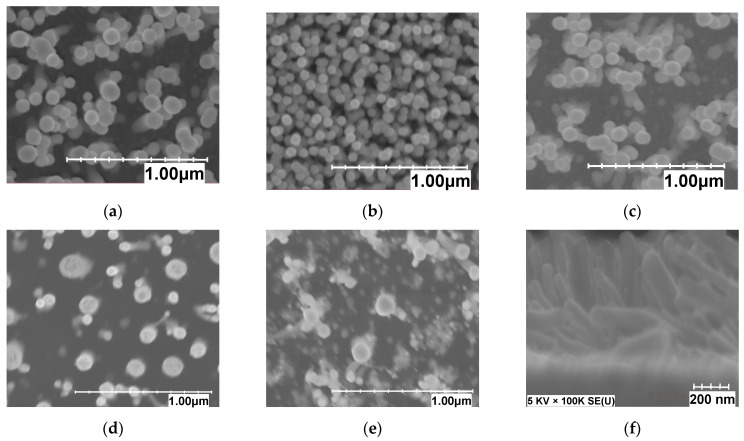
Fibers per area versus acetylene flow in sccm at 600 °C growth temperature and 9 nm nickel catalyst. (**a**) through (**e**) are top view SEM of the three samples and other flows not further investigated in the gas testing; (**f**) is a cross section view of sample (**e**). (**a**) 25 sccm. (**b**) 20 sccm (Sample A). (**c**) 15 sccm (Sample B). (**d**) 10 sccm (Sample C). (**e**) 5 sccm. (**f**) 5 sccm.

There are different ways to change the number of tubes per area in vertically aligned CNT such as varying the catalyst vapor concentration [26], pressure [27], time [28], and temperature [29]. In this study, we obtained different densities of fibers by changing the H_2_/C_2_H_2_ gas ratio in the CNF growth process [30], where H_2_ was fixed at 700 sccm. At a flow of about 5 sccm of acetylene gas, the nanofibers are not fully vertically aligned and intertwine. As shown in Figure 2, by increasing the gas flow up to 20 sccm, an increase in density was observed, and the tube alignment improved. For the gas sensing measurement, three different samples with different number of fibers per unit area were fabricated. The sample A with the highest density (about 8 × 10^10^ fibers/cm^2^) was obtained with 20 sccm of acetylene. For the samples B and C with lower densities (4 × 10^10^ fibers/cm^2^ and 5 × 10^9^ fibers/cm^2^, respectively), the results were obtained with 15 and 10 sccm of acetylene, respectively.

**Figure 3:**
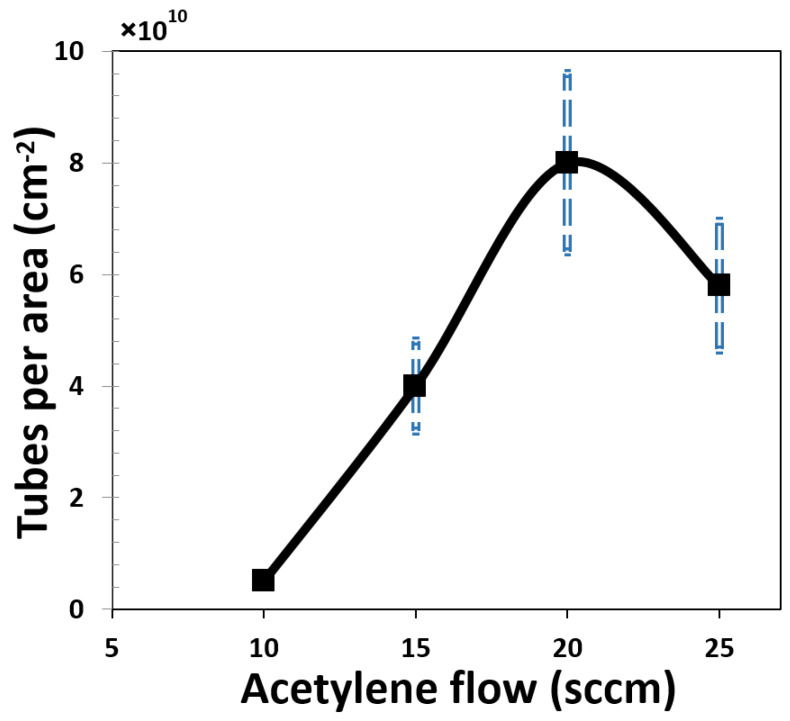
Number of fibers per area as function of acetylene flow.

The morphology of CNFs such as other nanostructure materials is critically important in the sensing mechanism because it can cause a greater absorption area and a favorable electronic structure [31,32]. The microstructures of the samples were probed by a scanning electron microscope (SEM; Hitachi, Regulus-8230). Figure 2 displays the obtained SEM images for the different densities. Figure 2a–e shows the top-view SEM images to demonstrate the effect of acetylene flow variations (in the rang 5–25 sccm) on the CNF density. Additionally, the cross-section SEM shown in Figure 2f indicates disordered, not-well-aligned fibers when using a 5 sccm C_2_H_2_ flow. Figure 2b–d is the top view of the three samples used in the gas sensing tests. The density was determined by counting the fibers per unit area. At least three deposition runs were performed for each density extraction. Additionally, to ensure accurate density estimates, different locations in each sample were examined. As can be seen, sample A had a very dense distribution of fibers and in sample C, fibers were clearly far apart. Figure 2e,f shows the effect of a sharp decrease in the flow of acetylene gas on the alignment of the fibers. The change in the number of fibers per unit area relative to the flow of acetylene gas is presented in Figure 3. The plot shows that increasing the acetylene flow to about 20 sccm led to an increase in density.

When the flow of acetylene is increased, meaning more carbon source is provided and the decomposition rate of the carbon source increases, this results in an oversupply of carbon in the form of amorphous and crystalline carbon. When the acetylene flow is too high (as a result, the ratio of H_2_/C_2_H_2_ is greatly reduced), the effect of hydrogen as the promoter of the catalyst is reduced. This phenomenon could reduce the production of clusters for fiber growth [33].

**Figure 4:**
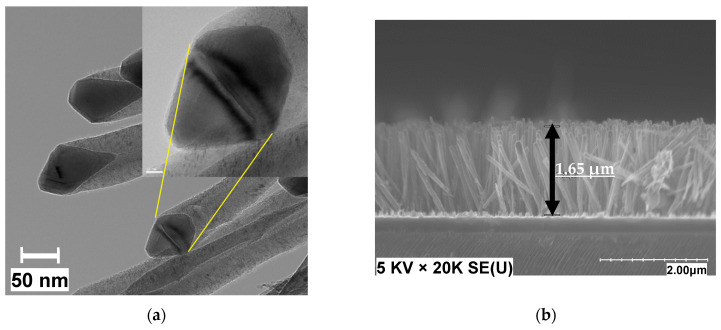
(**a**) TEM image of PECVD-grown CNF indicating a tip growth mechanism; (**b**) SEM cross-section image of CNF grown using 9 nm Ni at 600 °C.

To grow a uniform density of nanofibers (i.e., with similar length and diameters), we fixed the growth parameters including time, catalyst thickness, temperature, pressure, and plasma power in each sample. Transmission Electron Microscope (TEM; FEI, Tecnai G^2^) imaging was used to inspect the morphology of the CNF diameter [34]. Figure 4a shows a TEM image of CNFs that confirms that the filaments consist of CNF with a herringbone stacking of the crystal planes. It was also observed that the growth mechanism is tip growth. Using TEM, we measured a mean diameter of 50 nm ( ±5 nm) for this specific CNF. SEM cross section images were used to estimate the length of the fibers. As shown in Figure 4b, with a 20 min growth time in the presence of plasma, fibers with a length of about 1.65 μm were obtained.

**Figure 5:**
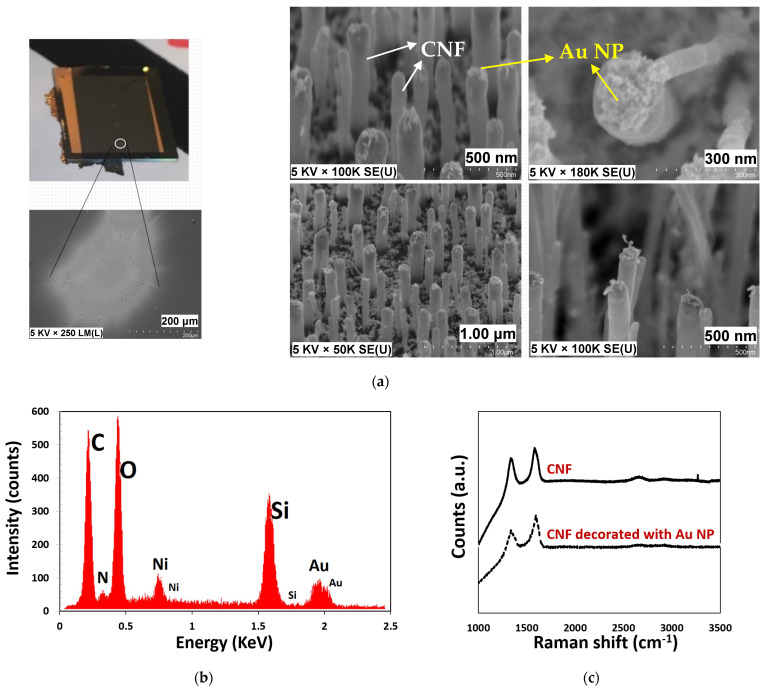
(**a**) Fabricated CNF sensor decorated with gold nanoparticles and zoomed-in view of printed nanoparticle point (left). SEM images of the CNF synthesized decorated with gold nanoparticles (right). (**b**) EDX spectrum of CNF with gold nanostructures. (**c**) Raman spectra of CNF and CNF decorated with gold nanostructures.

To increase the sensitivity of the sensors, gold nanoparticles (NP) were deposited on the CNF using aerosol impaction printing. SEM micrographs clearly show the presence of nanostructures on the surface of the nanofibers, Figure 5. As it is evident from Figure 5, NPs are mostly well dispersed on the top of the fibers due to the flow of particles coming from the top. The nanoparticle dimension was estimated to be less than 10 nm. This value matches other studies [35,36,37] that study the diameter of spark ablated NPs in depth. In addition, the decorated samples were examined by a Raman microscope (Renishaw, inVia) and with Energy Dispersive X-ray (EDX; FEI, Nova NanoSEM 450 with EDAX Octane detector), and the corresponding results are shown in Figure 5.

The presence of Au on the carbon nanofibers was confirmed by the EDX analysis results (see Figure 5b). From the EDX spectrum, there were no other metallic contaminants. The peaks for Si and O originated from the substrate, carbon, and gold, were found in the spectrum, while the Ni peaks come from the catalyst used for CNF growth.

We next performed Raman spectroscopy to investigate the change in phonon vibration of the decorating NPs on the CNFs [38]. CNF Raman spectra include the G-band, from the sp^2^ bonds in the CNFs, while disorder of any kind results in a D-band. Consequently, the ratio (I_D_/I_G_) band analysis showed the quality of CNFs, which was relatively low, which is not uncommon for CNF due to damage from the plasma during growth [39]. As shown in Figure 5c, Raman characterization did not show obvious structural changes on CNF supports after decoration. The I_D_/I_G_ value of the decorated CNF sample was calculated to be 0.39, whereas the value was 0.25 for the bare CNF. Additionally, slight shifts from 1579 to 1596 cm^−1^ occurred in the G-band on decorating CNF walls with Au NPs. We suspect an increase in the I_D_/I_G_ ratio occurs due to chemical bonding between Au and CNF, resulting in induced phonon stiffening. Besides, the electronic interaction and charge transfer between CNF and Au NPs causes a G-band shift [40].

### 3.2. Gas Sensing Measurements

**Figure 6:**
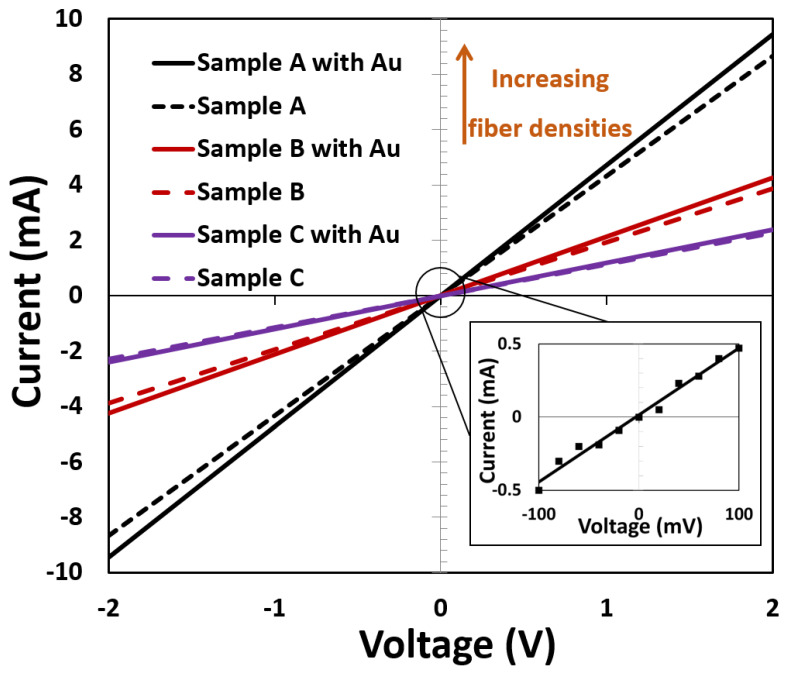
I–V characteristics of different densities of CNF-based sensor with and without Au NPs. The inset shows the I–V characteristic of Sample A with Au from −100 mV to +100 mV.

The output characteristics (I–V) of all different density CNF-based sensors before and after the decoration of Au NPs on the CNF are presented in Figure 6. All I-V curves show linear behavior in both positive and negative biased voltages, which indicates Ohmic contact before and after the decoration with Au NPs. Due to the sparse nature of the Au NP, they had only a small impact on the conductivity of the sample. Samples with higher densities had a higher conductivity, which was to be expected as the distance between tubes is reduced.

**Figure 7:**
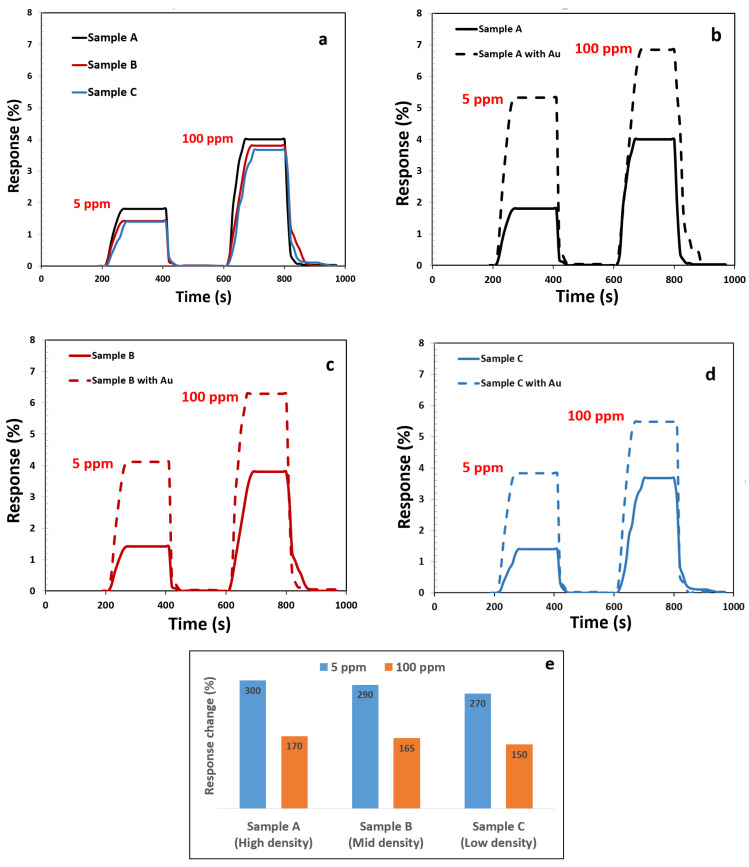
(**a**–**d**) Response of CNF and decorated at different concentrations of ethanol vapor at room temperature. (**e**) Percentage of response changes after decorated with gold.

Next, we illustrate how increasing the number of fibers leads to efficiently enhancing the gas sensing properties of the CNF-based gas sensor. For this, the gas sensing capabilities of three fabricated sensors exposed to different concentrations of ethanol vapor gas molecules were evaluated at the room temperature. The sample responses are depicted in Figure 7a. As can be seen, the conductivity of the sensors changed upon the exposure of gas molecules. The transfer of the weakly bonded electrons from ethanol molecules to the CNF surface changes the hole concentration in the CNF, giving rise to the change in the sensor resistances [41].

To investigate the effect of decoration, three sensors were also fabricated with the same growth condition and tested under the same experimental conditions after decoration. The gas sensing results are shown in Figure 7b for sample A and in Figure 7c,d for samples B and C, respectively. As is evident in Figure 7, increasing the density of CNF samples led to a small increase in the response to gas. The number of fibers per unit area is directly related to the surface-to-volume ratio in the CNF samples. Increasing the surface-to-volume ratio in CNF sensors enhances the gas absorption [42].

On the other hand, the presence of gold nanoparticles showed a considerable increase in the sensing capability of the decorated samples, in comparison with bare CNF sensors. As the plots in Figure 7a show, the response of the CNF sensors to 5 ppm and 100 ppm ethanol gas was about 1.8 and 4, respectively, at room temperature. On the other hand, the response of these samples when decorated with Au will increase up to 5 toward 5 ppm ethanol and 7 toward 100 ppm ethanol. The relative improvement was higher in sample A than in the other two samples. As it is clear from Figure 7e, the effect of decoration on gas sensitivity is greater at low concentrations, possibly due to saturation, so that, at the presence of 5 ppm ethanol gas, the decorated sample response was 300% more than the bare CNT.

The sensing mechanism of CNF-based gas sensors related to charge transfer between gas molecules and CNF surfaces. The low chemical reactivity of the CNFs with gas molecules is the main reason for the low sensitivity of the bare CNF at room temperature [43]. The CNF provides a template that NPs are absorbed and CNF surface link the NPs. Gold NPs link to the CNF surface via two mechanisms: (1) covalent bonds (2) or weaker intramolecular interactions such as π–π stacking and hydrophobic or electrostatic attractions [44]. In both cases gold NPs will increase the gas absorption sites on the surface, and this will enhance carrier transfer and cause a Fermi level shift [21]. In addition, the gold NPs can interact with the gas molecules and transfer positive charges to the CNF. This can greatly increase the response of the sensor at low temperatures, given that gold NPs themselves can act as gas sensors [45].

Sensor resistance increased in the presence of ethanol vapor (a reducing species), which confirms that the CNF behave as p-type semiconductors [46]. In addition, as Figure 6 depicted, Au NP has a minor effect on the conductivity of CNF sensors. Probably, adsorbed oxygen molecules on the Au NP act as extra carrier charges in the CNF surface; therefore, the number of carriers increases in the CNF sensors after decoration with Au [47]. When ethanol gas was introduced, the conductivity greatly decreased for the decorated CNF sensor. This can be explained by the removal of the adsorbed oxygen on the Au NPs by the ethanol molecules. Thus, the previously donated carriers from oxygens trapped by Au NPs reduce and, hence, decrease the conductivity. As Figure 7 shows, increasing CNF density causes increasing carrier transport and provides more sensitivity. From Figure 7a,e, it is clear that there is the dependence of the responsivity on the CNF density of the sensors. It is reasonable to assume that a high surface area supplies a larger number of surface-active sites for both oxygen adsorption [48] and surface reaction [49], resulting in a larger change in the surface properties of the CNF sensors and eventually increasing the gas responsivity.

Additionally, the response and recovery time of sensors were measured and recorded. The recovery time at different densities for the bare CNF sensor was almost constant and was recorded at about 35 s. However, the response time decreased with increasing fiber density. The response time of sample A was recorded as about 60 s, while in sample B, this time was recorded as 90 s. Meanwhile, gold decoration on the CNF sensors was found not to have a significant effect on the response and recovery time.

Discrete wavelet transforms (DWT) were employed in order to analyze the response signals of the gas sensors. DWT was used to ensure the same trend of the sensor dynamics response for different density sensors. The same sequence of numbers was obtained for each sample under different density, see also Figure S1. This characterization proves that the main gas sensing factor (nanofibers with graphene layers) is the same in all three types of CNF sensor with different densities. These DWT sequences are consistent with other references [41,50]. These results emphasize that another current path other than the fibers’ flow path, such as amorphous carbon or catalyst, had no effect on the measured resistance during gas sensing. However, further studies must be performed to elucidate the conduction path.

**Figure 8:**
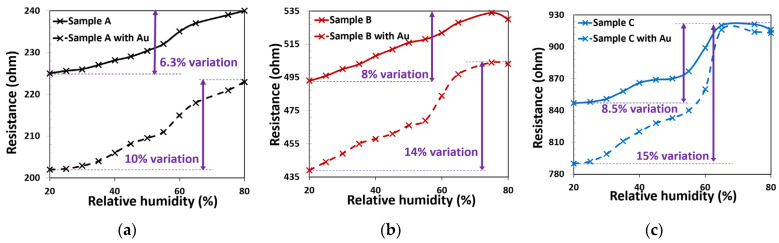
Variation in actual resistance of fabricated samples in terms of relative humidity at room temperature and atmospheric pressure. (**a**) Sample A and sample A decorated with Au NPs. (**b**) Sample B and sample B decorated with Au NPs. (**c**) Sample C and sample C decorated with Au NPs.

We also analyzed the effect of temperature and humidity on the sensitivity of the device for different concentrations of ethanol vapor. The variation in humidity changes the conductance of the fabricated sensors. In the latest research of our group, it has been reported that humidity changes have effects on the gas sensing properties of the CNFs [51]. Although the gas response of CNF sensors increases at higher CNF densities, the response to increasing relative humidity decreases. This is illustrated in Figure 8, where the conductivity to the variation of relative humidity reduces by 2.2% percentage points when going from samples C to A. Therefore, the effect of humidity on the gas sensing property of the high-density CNF sample is lower than samples B and C. Water molecules can form a layer around the tubes adding a parallel resistance. This resistance changed the upward trend of the resistance of the samples in high relative humidity, as can be observed in Figure 8b,c. This phenomenon has also been observed in our previous study [51].

CNF decorated with Au Np sensors behave similarly to CNF sensors in a variation of humidity. Likewise, increasing the humidity in these sensors reduces the conductivity. As can be observed in Figure 8, decreasing the density from sample A to sample C led to a 5% increase in conductance variation in decorated CNF sensors. Additionally, Figure 8 illustrates that decorated CNFs show a larger sensitivity than intrinsic CNF toward relative humidity that is the same as sensitivity to ethanol vapor.

**Figure 9:**
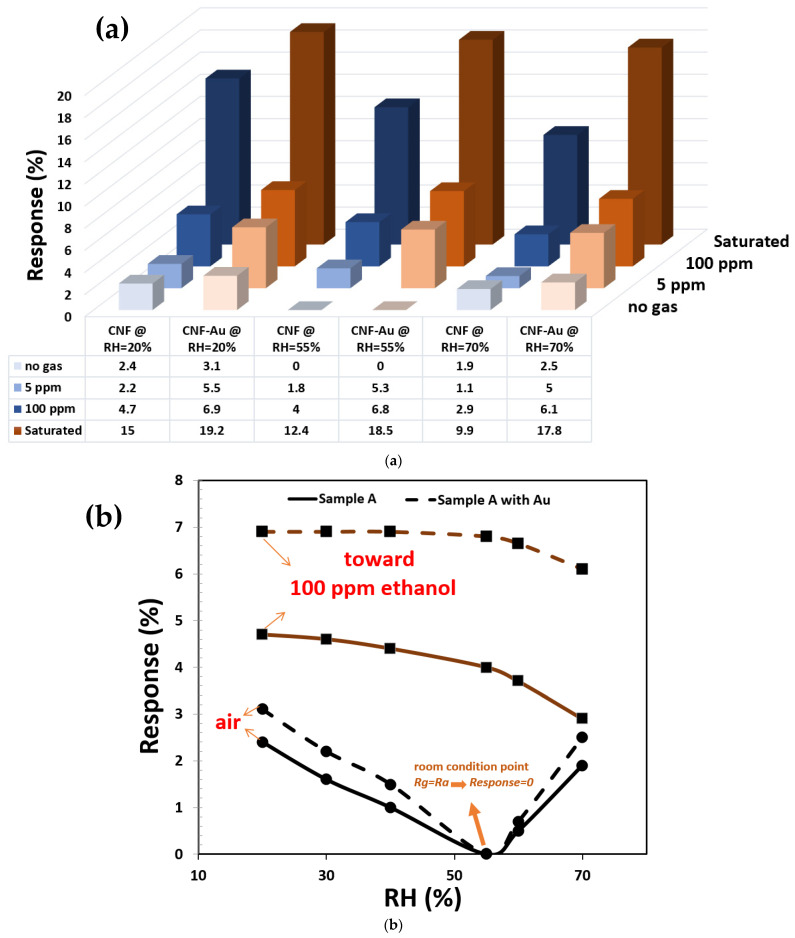
(**a**) Response of CNF-based sensor in presence of different concentrations of ethanol vapor and different variation of relative humidity at room temperature (the blue and brown spectrum represent CNF and decorated CNF, respectively). (**b**) Response of sample A in air and presence of 100 ppm concentration of ethanol vapor toward variation in relative humidity at room temperature.

Figure 9 shows that the effect of humidity on the gas sensing response to ethanol was lower in decorated samples. This phenomenon may be due to several reasons: (1) humidity changes impure oxygen on the CNF surface. With an increase in the surface-to-volume ratio—due to the increasing number of fibers—the concentration of H_2_O molecules does not affect the number of oxygen molecules. (2) The increase in water molecules can act as a parallel resistance on the surface of CNFs, while in high-density CNFs density this resistance rarely occurs. (3) Type changing in CNFs in the presence of humidity results in the type of carrier transfer changes between the CNF and water molecules.

**Figure 10:**
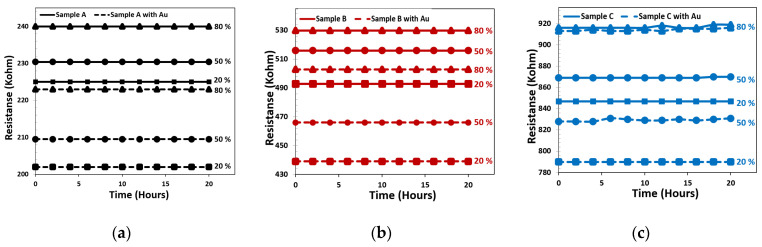
The short-term stability of CNF-based sensors, measured at 1V at various RH levels.

The stability of CNF and decorated CNF sensors was investigated in different constant relative humidity. The sensing results were carried out at room temperature every hour for three times, with RH ranging from 20% to 80%. Figure 10 shows the effect of short-term stability for two sensors. The sensors’ conductance had no obvious deviation for CNF and decorated CNF. From the data of Figure 10, it can be understood that the coefficient of variation of the resistance change (stability index) in sample A was less. So, it can be concluded that increasing the number of fibers per unit area leads to greater stability to relative humidity. Variation in the resistance change in RH = 80% was 0.11%, 0.28%, and 0.42% for samples A, B, and C, respectively.

**Figure 11:**
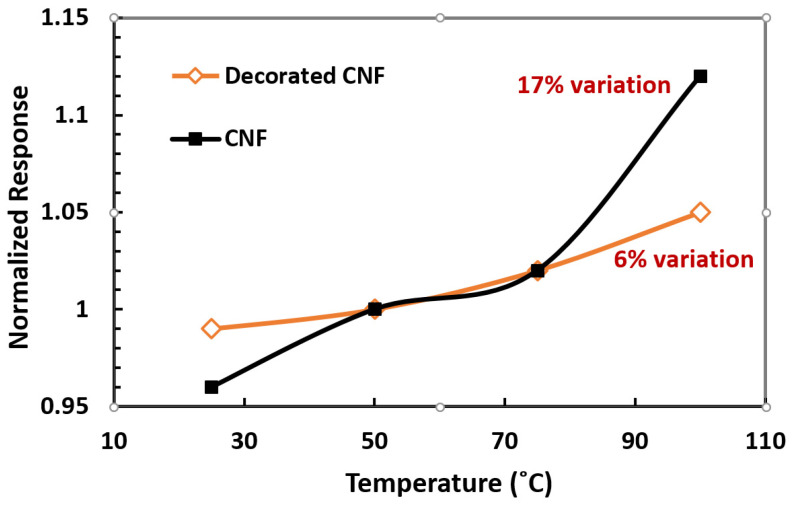
Variations in response levels of CNF and decorated CNF samples by changing operation temperature range at the presence of saturated concentration of ethanol vapor.

The thermodynamic of gas adsorption that is related to temperature affects CNF gas detection. The conductivity of the CNF devices decreases by increasing the temperature to 100 °C. As shown in Figure 11, when exposed to ethanol vapor at elevated temperatures, the CNF response to analytes again increases. Increasing the temperature promotes more adsorbed oxygen species to participate in the reaction, thereby improving the response [52,53,54]. As shown in Figure 11, a NP-decorated CNF with gold experiences lower sensitivity to the temperature during the gas sensing mechanism. So, the response of a decorated CNF sample in the presence of a saturated concentration of ethanol vapor had a 6% variation with a 75 °C change, while in the bared CNF, it had a 17% variation in response to the same temperature change. The resistance measured across the CNF-resistive sensor is the sum of the following contributions: the electrical resistance offered by the fibers to electron flow, which decreases with temperature, and the electrical barrier offered by metal-CNF contact (caused by both NPs and electrodes). The first term depends on the interaction of the CNF surface with the oxidizing/reducing gases and dominates over the second term in the temperature range of operation. This reaction occurring on the surface of the CNF is a thermally activated process and hence increases with temperature. Additionally, the charge carriers in the CNF are temperature-dependent [55]. However, the second term, although it changes with increasing temperature, has no effect on the interaction with the gas molecule. Therefore, in decorated samples, the effect of temperature in response to gas is less.

**Figure 12:**
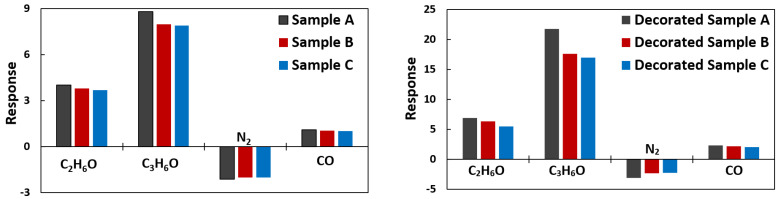
Selectivity measurements of the three different density samples, comparing the response toward ethanol, acetone, nitrogen, and carbon monoxide gases. In the measurements, concentration of different gases is fixed at 100 ppm at room temperature.

Moreover, all samples toward ethanol and other gases were also tested to investigate the effect of the number of fibers per area and decoration on the selectivity. From Figure 12, the low selectivity of the proposed samples is vividly clear. Nevertheless, both CNF and decorated CNF work better as an acetone sensor. The selectivity was studied by keeping the gas dose constant at saturated concentrations but varying the gas type. The presence of positive as well as negative responses in Figure 12 can be seen. CNF + O_2_ and CNF + AuNPs + O_2_ makes the combination capable of sensing both electron-donating (positive response) and electron-accepting (negative response) gases [56]. All tested gases endure chemisorption processes except nitrogen gas, which exhibits physisorption [57]. N_2_ interacts hardly even with AuNPs as a catalyst because the triple bond N-N is so strong and stable. It probably removes other surface contaminants instead of reacting itself with the CNF.

## 4. Conclusions

In this study, three different structures with different densities of PECVD-deposited CNFs were fabricated for ethanol gas sensing applications. To enhance the gas sensing, aerosol impaction printing has been used to deposit gold nanostructures on the CNF surfaces. The structures of the fabricated samples were examined by SEM and TEM micrograph, Raman spectroscopy, and an EDX pattern, by which the correct growth of CNFs and the formation of the gold nanoparticles was confirmed. From the results obtained in this study, we conclude that the high-density CNF with Au NPs decoration sensor sample operated at room temperature exhibit an increased sensing response toward ethanol vapor, especially in low ethanol concentrations. Moreover, we found that the change in humidity and temperature has less effect on the gas response on decorated samples having high density and does not significantly alter sensing of the ethanol gas. Other decoration strategies must be considered to overcome the low selectivity of the fabricated sensors.

## Data Availability

The data presented in this study are available from the corresponding authors upon reasonable request.

## Supplementary Materials

The following are available online at https://www.mdpi.com/article/10.3390/ma15041383/s1, Figure S1: Results extracted of DWT decomposition for gas response value of different density CNF sensor to 100 ppm of ethanol vapor.
