# Supplementary material for: Enhancement of Room Temperature Ethanol Sensing by Optimizing the Density of Vertically Aligned Carbon Nanofibers Decorated with Gold Nanoparticles
Source: Materials (Basel). 2022 Feb 13;15(4):1383. doi: 10.3390/ma15041383 (PMC8879461; doi:10.3390/ma15041383)
Supplement: Supplementary file 1 [file materials-15-01383-s001.zip › materials-1581626-supplementary.pdf]

Enhancement of room temperature Ethanol sensing by optimizing the density of Vertically Aligned Carbon Nanofibers decorated with Gold nanoparticles

Mostafa Shooshtari[1], Leandro Nicolas Sacco[2], Joost van Ginkel[2], Sten Vollebregt[2] and Alireza Salehi[1]

[1] Department of Electrical Engineering, K N Toosi University of Technology, Tehran, Iran
[2] Department of Microelectronics, Laboratory of Electronic Components, Technology and Materials (ECTM), Delft University of Technology, Delft, CD, The Netherlands

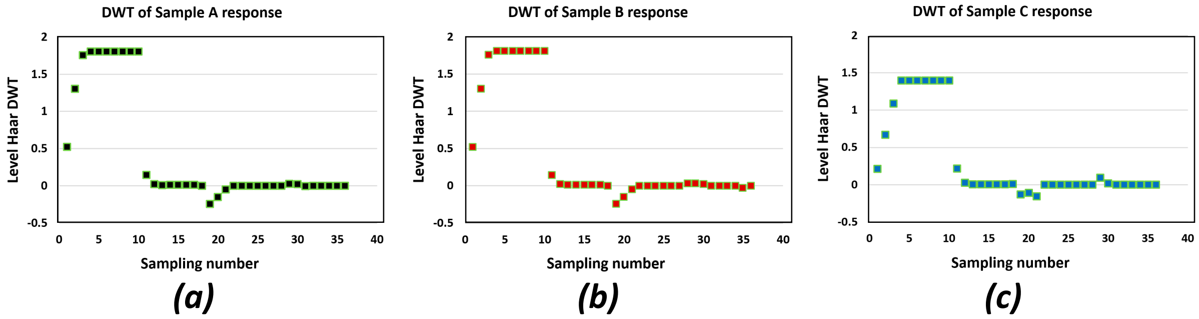

Figure S1: Results extracted of DWT decomposition for gas response value of a) high-density (sample A), b) Mid-density (sample B) and c) low-density (sample C) CNF sensor to 100 ppm of ethanol vapor.
